# Mental Health during COVID-19 Pandemic: The Role of Optimism and Emotional Regulation

**DOI:** 10.3390/ijerph19031413

**Published:** 2022-01-27

**Authors:** Imen Krifa, Llewellyn Ellardus van Zyl, Amel Braham, Selma Ben Nasr, Rebecca Shankland

**Affiliations:** 1 Université de Sousse, Faculty of Medicine of Sousse, Mental illness Epidemiology Research Laboratory LR12ES04, Screening and Early Management, Sousse 4000, Tunisia; krifa_imen@yahoo.com (I.K.); braham.amel@yahoo.fr (A.B.); selmabennasr@yahoo.fr (S.B.N.); 2 Université de Sousse, Higher School of Sciences and Techniques of Health of Sousse, Sousse 4054, Tunisia; 3 Department of Industrial Engineering & Innovation Sciences, University of Eindhoven, 5612 Eindhoven, The Netherlands; l.e.v.zyl@tue.nl; 4 Optentia Research Focus Area, North-West University (VTC), Vanderbijlpark 1900, South Africa; 5 Department of Human Resource Management, University of Twente, 7522 Enschede, The Netherlands; 6 Department of Social Psychology, InstitutfürPsychologie, Goethe University, 60323 Frankfurt, Germany; 7 Farhat Hached University Hospital of Sousse, Department of Psychiatry, Sousse 4000, Tunisia; 8 Laboratory DIPHE (Development, Individual, Personality, Handicap, Education), Department of Psychology of Development, Education and Vulnerabilities, University Lumière Lyon 2, 69676 Bron, France; 9 Laboratory LIP/PC2S, Department of Psychology, University Grenoble Alpes/Savoie-Mont-Blanc, 38000 Grenoble, France

**Keywords:** COVID-19, emotional regulation, optimism, study engagement, anxiety, depression

## Abstract

In light of different challenges associated with the COVID-19 pandemic, university students are considered a particularly vulnerable population to mental health and study engagement issues. The first years at university represent a crucial period for students and are associated with an increase in mental health problems, particularly in healthcare studies. This study aimed (1) to document the current levels of mental health and study engagement among healthcare students in Tunisia, and (2) to investigate the relationships between emotional regulation, optimism, study engagement and common mental health problems (stress, anxiety and depression) among this population. A cross-sectional, electronic survey-based research design was used to draw a sample of 366 health care students from a University in Tunisia. Participants mostly reported mild (34.7%) or moderate (44.3%) levels of depression, moderate (44.7%) or severe (33.6%) levels of anxiety, average (50.8%) or mild (33.8%) levels of stress, and high levels of study engagement (>85%). Through structural equation modelling, the results showed that emotional regulation negatively affected stress, anxiety, and depression. Optimism partially mediated the relationship between emotional regulation, anxiety and depression and fully mediated the relationship between emotional regulation and study engagement. The findings indicated a high prevalence of psychological distress among healthcare university students in Tunisia, and specific protective factors that may be targeted to reduce mental health problems.

## 1. Introduction

As a public health emergency of international concern, COVID-19 has gained intense attention since its spread began in December 2019 [1]. In January 2020, the World Health Organization (WHO) declared the emergence of COVID-19 as a major public health emergency of worldwide concern [2]. When it is unfamiliar, highly contagious and fatal, such as with the COVID-19 virus, a variety of psychological problems have been common reactions to the pandemic, which may lead to mental health problems [3].

In light of the different challenges associated with the current COVID-19 pandemic, university students are considered a population vulnerable to the onset of common mental health problems such as stress, depression and anxiety [4,5]. These could negatively affect their social interactions, motivation, concentration and study engagement [6]. Research suggests that university students are three times more likely to develop mental health problems than the general population [7], and that experiencing psychological distress is a common problem in higher education [8]. University students are particularly vulnerable to the onset of common mental health problems and psychological distress compared to the age-matched general population [9].

The first years at university represent a crucial period for students’ mental health and growth, which requires using active coping strategies to best adapt to this new environment [10]. Early adulthood represents a high-risk period during which different psychological disorders may appear [7]. This results from new responsibilities and challenges that young adults face when adapting to university life [11]. In fact, this transition brings a host of new stressors such as leaving home, developing one’s autonomy in daily tasks, new contexts without well-known friends or parental close social support, new social relationships to build, as well as academic pressure and decision-making challenges (e.g., Baghurst et al. [12]). University students report loneliness and interpersonal relationship conflicts while struggling to manage their time effectively because of a heavy curriculum [13]. These factors can lead to academic burnout, which reduces their ability to cope with university life’s different responsibilities [13,14]. Students report experiencing high levels of stress, which can have serious consequences (e.g.,Houghton et al. [15]). These stressors affect students’ normal day-to-day activities and lead to sleep disorders and lower academic performance, which in turn may lead to study dropout, social isolation, substance abuse, reduced life satisfaction, loss of self-confidence, and suicidal ideation [12].

Higher levels of stress and academic burnout have been widely documented among medical trainees and nurse students [14,16,17,18,19]. Clinical training is a significant component of students’ professional development as medical practitioners but can also be highly stressful [20,21]. During this time, students are faced with various stress-inducing challenges ranging from maintaining positive interpersonal relationships with clinical staff and instructors, managing patient demands, and determining how to manage sudden changes in patients’ conditions as well as becoming used to witnessing suffering, death and being constantly observed and evaluated [22,23]. These clinical experiences are crucial for their professional development but may increase stress and anxiety [22]. These high levels of stress may in turn cause psychological impairment that may result in lower levels of confidence in their own professional abilities and negatively affect the quality of the care they provide [24].

In certain countries, levels of stress have been reported to be higher than in other parts of the world. This is the case of Tunisia, where healthcare students’ levels of psychological distress have shown to be significantly higher than those in other countries [25], with 74.1% of the participants who reported definite (43.6%) or probable (30.5%) anxiety symptoms, and 62% who reported definite (30.5%) or probable (31.5%) depression symptoms, while the levels of depression reported by a meta-analysis performed on 54 studies carried out among resident physicians in other countries reported a prevalence of depression which varied from 3 to 60% with a median of 28.8% [19]. Among Tunisian healthcare students, the levels of anxiety and depression were related to workload: number of hours worked per week (median = 60 h), and number of nightshifts per month (median = 6) [25]. Furthermore, the authors indicated that medical residents in Tunisia face particularly difficult work situations, as they are the only physicians present in hospitals at night, on weekends and on holidays. The high workload and high responsibility levels may thus represent specific risk factors for healthcare students.

In such populations, the current pandemic may represent a further stress factor, which may increase levels of anxiety and depression, and lead to reduced study engagement. As it may currently be difficult to act upon the risk factors, it may be more effective to tackle mental health protection factors. Therefore, specific protection factors should be identified in order to develop mental health promotion programs that target these specific factors. Mental health promotion interventions may be particularly relevant for university students, especially in specific fields which have been identified as generating higher levels of stress and anxiety because of greater study demands.

In order to develop such interventions, it is necessary, as a first step, to study the specific characteristics of the healthcare students who are likely experiencing psychological distress, and to identify potential protective factors in this specific population. Several protective factors have been identified that enable the enhancement of student wellbeing, and buffer the effects of risk factors on student stress and psychological distress [26]. These protective factors are particularly effective during the current pandemic, with higher levels of social support (peer and teacher) being able to buffer against mental health issues [4,5]. Further buffering resources also include student characteristics such as emotional regulation and optimism [26,27].

Emotion regulation can be defined as the processes by which we influence the valence and intensity of emotions [28]. Emotional regulation enables one to develop and maintain positive relationships, thereby contributing to increased social support [29,30,31], which has been shown to be an essential protection factor for student mental health [32,33]. Furthermore, a recent systematic review showed that emotional regulation helped recover faster from acute stress [34]. This may imply that during the COVID-19 pandemic, such a protection factor could be particularly important to develop [35].

Gross’s process model of emotion regulation is the most commonly used framework to study emotion regulation strategies [36,37]. In adults, difficulties in emotional regulation are associated with greater mental health problems (e.g., alcohol abuse, anxiety and mood disorders) [36]. Conversely, emotional regulation has been shown to reduce risks of mental health problems and involves both increasing the frequency of positive emotional experiences and reducing the frequency of negative emotional experiences [38].

Positive emotions broaden the individual’s thought and action repertoires, thereby enhancing more diverse, flexible, and creative thoughts, as well as enhancing openness to new experiences [39] and resilience in response to stressful situations [40]. Furthermore, these broadened thought and action repertoires can build enduring personal and social resources that contribute to one’s resilience in the face of adversity [40]. Positive emotions may thus help increase a general positive mindset characterized by greater optimism.

Optimism is a disposition which refers to individuals’ generalized positive expectations about their future [41]. Research has shown that optimistic individuals are positive about different events in daily lifeand more confident regarding goal success, which was associated with lower levels of psychological distress [42,43]. Optimistic beliefs have been shown to be useful when confronted with difficulties and stressful life circumstances [44], are associated with problem-focused coping strategies that lead to greater resilience to stress, and with the tendency to view life’s stressors and difficulties as temporary and external to oneself [45]. Optimism has also been shown to predict student mental health and wellbeing [11,27,44]. Since Scheier and Carver’s seminal work, further research has found that optimistic university students were both physiologically and psychologically healthier than pessimistic students [26,41].

Hence, both emotional regulation and optimism have been shown to affect physical and psychological health, but studies are needed to show how optimism mediates the relationship between emotional regulation and mental health outcomes. Therefore, it could be hypothesized that emotional regulation (i.e., negative emotions down-regulation and positive emotions up-regulation) would lead to a more positive and optimistic mindset, which in turn would reduce common mental health problems. Furthermore, this positive mindset may also lead to positive educational outcomes such as academic motivation and increased levels of study engagement [46].

The concept of study engagement originated from the work engagement model developed by Schaufeli et al. [47], and refers to a positive, fulfilling, work-related state of mind characterized by vigour, dedication, and task-related absorption.This construct was then applied to the academic tasks students perform [48]. Vigour refers to high levels of energy, mental resilience and willingness to invest effort in studying. Dedication refers to being strongly involved in one’s study-related tasks and experiencing a sense of significance and enthusiasm while working. Absorption refers to being fully concentrated in study-related tasks, experiencing that time passes quickly and that it may be difficult to detach from studying [49].

Throughout the past decade, study engagement has been shown to be one of the most integral components needed for students to feel good, function well and fit in to university life [49]. When medical students are actively engaged in their studies, it should lead to positive individual (e.g., mental health, wellbeing) and study-related outcomes (e.g., academic performance) [50,51]. Specifically, it could aid students in experiencing lower levels of stress, depression and anxiety at university [46].

The aim of the current study was thus twofold: first to document levels of mental health and study engagement among Tunisian healthcare students during the development of the COVID-19 pandemic, and second, to analyse the relationships between these variables in order to analyse how emotional regulation and optimism affect mental health and study engagement during this period. More specifically, based on the broaden-and-build model of positive emotions, we hypothesized that emotional regulation and optimism would be related to lower levels of stress, anxiety and depression, and higher study engagement levels.

## 2. Materials and Methods

### 2.1. Research Approach and Procedure

A cross-sectional research design was used to determine the relations between emotional regulation, optimism, study engagement and common mental health problems (stress, anxiety, and depression). Data was collected from the Higher School of Sciences and Techniques of Health at Sousse in Tunisia, at the beginning of 2021 (January–March). The Tunisian ethics committee at the university of medicine of Sousse gave ethical clearance. Subsequently, written permission was given from the high school authority. The purpose of the study was explained to the students, and written consent was obtained from each participant.

### 2.2. Participants and Sampling Strategy 

A total sample of 366 healthcare science students (out of 412 students) from a high school in Sousse in Tunisia took part in the study. Table 1 provides an overview of the demographic characteristics of the sample, and of their mental health and study engagement levels. The majority of the participants of this study were single (98.6%), females (94%), without any child (98.6%), living in a student residence (44%). Most participants were in their first year (38.8%) of an Emergency Care (31.1%) degree, and had never repeated an academic year (99.2%).

### 2.3. Measures

The following instruments were administered during the study. A demographic questionnaire was used to collect information about individuals’ gender, marital status, parental status, study field, year of enrolment, and living arrangements.

The Emotional Self-Regulation Sub-scale of the French Profile of Emotional Competence was used to measure overall emotional regulation (PEC [52]). This 5-item subscale measures emotional regulation on a 5-point Likert scale, ranging from 1 (totally disagree) to 5 (totally agree). An example item is, “When I am angry, I find it easy to calm myself down”. The emotional regulation subscale of the PEC has shown to be a valid and reliable measure with Cronbach’s Alphas ranging from 0.60 to 0.88 [52].

The French version of the Life Orientation Test-Revised (LOT-R) was used to evaluate the disposition towards optimism [43]. The revised version comprises 10-items rated on 5-point Likert scale ranging from 0 (strongly disagree) to 4 (stronglyagree). Five of these items measure dispositional optimism, whereas the rest are filler items. An example item reads, “If something can go wrong for me, it will”. The LOT-R has shown to be a valid and reliable measure in various contexts, with Cronbach’s Alphas ranging from 0.63 to 0.83 [53].

The French version of the 2006 Utrecht Work Engagement Scale for students (UWES-9S) developed by Schaufeli et al. was used to measure study engagement [54]. The nine-item questionnaire is rated on a six-point Likert scale ranging from 0 (Never) to 6 (Always). It measures the three components of study engagement with three items each. Example items are, “When I am doing my work as a student, I feel bursting with energy” (Vigour), “I am proud of my studies” (Dedication), and “I get carried away when I am studying” (Absorption) [54]. For the Vigour subscale, normal scores range from 3.26 to 4.80, while high and very high scores range from 4.81 to 5.65 and ≥5.66. For the Dedication subscale, the normal scores range between 2.91 and 4.70, while high and very high scores range from 4.71 to 5.69 and ≥5.70. For the Absorption subscale, the normal scores range from 2.34 to 4.20, while the high and very high scores range from 4.21 to 5.33 and ≥5.34 [55]. The UWES-9S has shown to be a valid and reliable measure in various contexts, with Cronbach’s Alphas ranging from 0.72 to 0.93 [54].

The French version of the Depression, Anxiety and Stress Scale-21 (DASS-21) was used to measure students’ common mental health problems [56]. The DASS-21 is composed of 21 self-report items that measure depression (e.g., “I found it difficult to work up the initiative to do thing”), anxiety (e.g., “I felt I was close to panic”), and stress (e.g., “I found it difficult to relax”) with seven items each. Each item is rated on a 4-point Likert-type scale ranging from 0 (did not apply to me at all) to 3 (applied to me very much or most of the time). Normal scores for the depression subscale range from 0 to 9, while pathological scores range from 10 to 28. For the anxiety subscale, the normal scores range from 0 to 7, while pathological scores range from 8 to 20. For the stress subscale, normal scores range from 0 to 14, while the pathological scores range from 15 to 34 [57]. The DASS-21 has shown to be a reliable instrument in various contexts with Cronbach’s Alphas ranging from 0.72 to 0.97 on the various subscales [56].

### 2.4. Statistical Analyses

Both SPSS v26 [58] and Mplus v 8.4 [59] were used to process the data. A three-phased analytical strategy through structural equation modelling was used to investigate the relationship between the factors. First, descriptive statistics (ito means, standard deviations, Skewness and Kurtosis) were produced to determine multivariate normality and composite reliability estimates to determine the internal consistency of the various instruments, and Pearson correlations were used to explore the relationships between factors (*p* ≤ 0.01). Skewness and Kurtosis between −2 and +2 were indicative of multivariate normality [60]. Point-estimate composite reliability (upper-bound; ρ > 0.70) [61] was computed to determine the internal consistency of the various scales.

Second, within the structural equation modelling approach, a competing confirmatory factor analytical (CFA) measurement modelling strategy was employed to determine the best-fitting model for the data. The robust weighted least square mean and variance adjusted (WLSMV) estimator in Mplus was used to provide a better option for modelling categorical data [62]. Based on a priori CFA structures, five competing measurement models were estimated and sequentially compared to determine the best-fitting model for the data. The best-fitting measurement model was determined through both traditional model fit statistics [63], and measurement quality [64]. Initially, the Hu and Bentler (1999) model fit criteria were used to determine the data-model fit and discriminate between the different models (c.f. Table 2 for the Model Fit Criteria) [63]. Thereafter, measurement quality was determined through inspecting the standardized factor loadings (λ > 0.40; *p* < 0.05), item uniqueness (δ > 0.10 but <0.9; *p* < 0.01), and the presence of multiple cross-loadings. A model needed to show both excellent model fit and measurement quality to be retained for further analyses [64].

Third, a structural model was specified based on the best fitting measurement model. Where the measurement model allowed for covariance structures between latent variables (represented by observed indicators), the structural model aimed to estimate the directionality of the relationship between latent factors. Model fit was determined through conventional standards (c.f. Table 2). Items were permitted to be freely estimated, and no extra constraints were placed on the model. Relationships were determined through statistically significant standardized beta coefficients (*p* < 0.05).

Finally, to determine the indirect effects between the exogenous and endogenous factors, Preacher, Zyphur and Zhang’s, 2011 bias-corrected bootstrapping (BCB) method was used [65]. Here, a 10,000 BCB was imputed in order to determine the 95% confidence interval (CI 95) limits and standard errors for the indirect effect assessment. The significance for the indirect effect estimate was set at *p* < 0.05 and the 95% CI should not include zero [65].

## 3. Results

The descriptive statistics, competing measurement models, structural model and indirect effects are reported separately in this section. The results are tabulated and accompanied by a brief interpretation.

### 3.1. Descriptive Statistics, Internal Consistency and Pearson Correlation Coefficients

The descriptive statistics, internal consistency (composite reliability) and Pearson’s correlations are summarized in Table 3. The results showed that the data were normally distributed (Skewness and Kurtosis range between −2 and 2). With the exclusion of the Absorption subscale and optimism, scales showed high levels of upper bound composite reliability (upper-bound; *ρ* > 0.70; [61]). Furthermore, all factors were statistically significantly related (*p* < 0.05). The results showed mild to severe levels of mental health problems during the COVID-19 pandemic spread in Tunisia healthcare students. Conversely, a vast majority of students reported high or very high levels of study engagement.

### 3.2. Competing Measurement Models

Next, a series of theory-informed competing confirmatory factor analytical models were estimated to determine the data’s best-fitting model. Here, observed indicators (measured items) were treated as indicators for first-order latent factors. To ensure convergence, the factor variance for Study Engagement was fixed to 1 and the factorial indicators for Vigour, Dedication and Absorption were constrained to be equal. Furthermore, three items (Vigour_1 = “When I am doing my work as a student, I feel bursting with energy”, Emotional Regulation_3 = “I find hard to manage my emotions”, and Optimism_3 = “If something can go wrong for me, it will”), were removed from all analyses due to non-significant factor loadings. Items and error terms were permitted to be freely estimated. No items were parceled, nor error terms permitted to be correlated in order to improve model-fit.

As such, five measurement models were computed and compared:

Model 1: Optimism, Emotional Regulation, Stress, Depression, and Anxiety were estimated to be single, first-order factorial models. Engagement was estimated as a second-order factorial model comprised of three first-order factors: Vigour, Dedication and Absorption.

Model 2: All factors were specified as single, first-order factors with items loading onto their respective a priori theoretical factors.

Model 3: Optimism, Emotional Regulation, and Engagement were estimated to be single, first-order factorial models. Stress, Depression and Anxiety were estimated to be three first-order factors that led to a second-order factorial model called “Common Mental Health Problems”.

Model 4: Engagement was estimated as a second-order factorial model comprised of three first-order factors: Vigour, Dedication and Absorption. Stress, Depression and Anxiety were estimated to be three first-order factors that led to a second-order factorial model called “Common Mental Health Problems”. Optimism and Emotional Regulation were specified to be single first-order factors.

Model 5: Stress, Depression and Anxiety were estimated to be a bi-factor model, with “Common mental health problems” as the General factor, and Stress, Depression and Anxiety as three independent specific factors. Engagement was estimated as a second-order factorial model comprised out of three first-order factors: Vigour, Dedication and Absorption. Optimism and Emotional Regulation were specified to be single first-order factors.

Table 4 provides a summary of the overall model fit statistics of each competing measurement model. The results showed that only Model 1 (χ^2^_(649,*n*=366)_ = 968.72; CFI = 0.93; TLI = 0.93; RMSEA = 0.04 [CI: 0.032–0.041]; SRMR = 0.05) met the model fit criteria. In respect to measurement quality, Model 1 showed acceptable standardized factor loadings (*λ* > 0.40; *p* < 0.01), standard errors, and item uniqueness (*δ* < 0.10 but >0.9; *p* < 0.01) for all but two items; thus meeting the classification criteria [66,67]. Two items on the Optimism Scale (Optimism_7: *λ* = 0.25 and Optimism_9: *λ* = 0.27) did not meet the 0.40 factor loading threshold. These items were then removed and the model re-estimated.

The adapted version of Model 1 showed similar levels of model fit (χ^2^_(578,*n*=366)_ = 874.364; χ^2^/*df* = 1.49; CFI = 0.93; TLI = 0.93; RMSEA = 0.04 [CI: 0.032–0.041]; SRMR = 0.05). A chi-square difference test in Mplusshowed no statistically significant differences between Model 1 with or without these two Optimism items. Furthermore, the Optimism scale’s composite reliability only slightly increased (from ρ = 0.61 to ρ = 0.63) when removing these items. Model 1, including the two Optimism items, was therefore retained for further analyses.

### 3.3. Estimating the Structural Model

**Figure 1:**
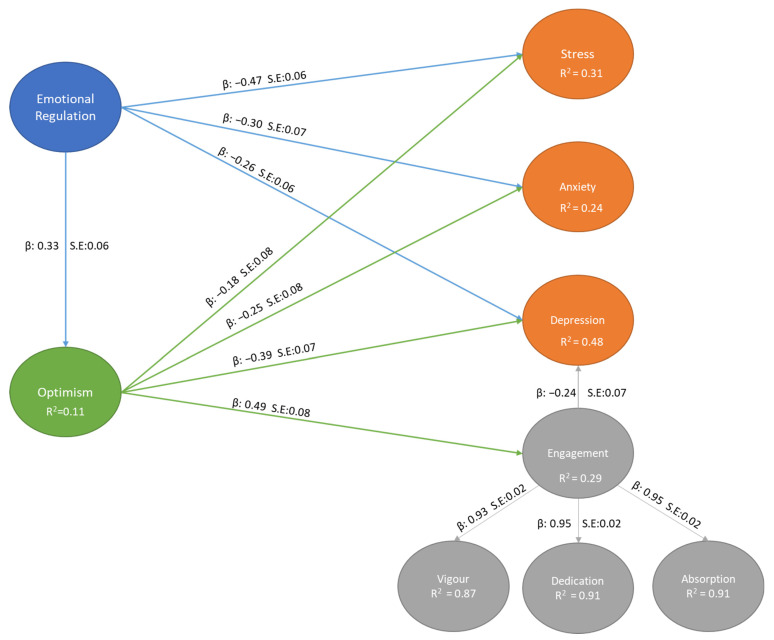
Structural Model.

A structural model was therefore specified based on the best fitting measurement Model 1 (c.f. Figure 1). Measurement Model 1 showed to be the most parsimonious and most accurately represented our data. The structural model showed acceptable levels of model fit (χ^2^_(649,*n*=366)_ = 968.72; χ^2^/*df* = 1.49; CFI = 0.93; TLI = 0.93; RMSEA = 0.04 [CI: 0.032–0.041]; SRMR = 0.05).

The results showed that Emotional Regulation negatively related to Stress (*β*: −0.47, S.E.:0.06, *p* < 0.05), Anxiety (*β*: −0.30, S.E.:0.07, *p* < 0.05), and Depression (*β*: −0.26, S.E.:0.07, *p* < 0.05). Similarly, Optimism showed to also negatively relate to Stress (*β*: −0.18, S.E.:0.08, *p* < 0.05), Anxiety (*β*: −0.25, S.E.:0.08, *p* < 0.05), and Depression (*β*: −0.39, S.E.:0.07, *p* < 0.05). Furthermore, Study Engagement was only negatively associated with Depression (*β*: −0.24, S.E.:0.07, *p* < 0.05). Cumulatively, both Emotional Regulation and Optimism comprised31% of the Variance in Stress, 24% in Anxiety, and 48% in Depression.

Furthermore, Emotional Regulation showed to be positively associated with optimism (*β*: 0.33, S.E.:0.06, *p* < 0.05), and comprised 11% of the total variance therein. However, in the presence of Optimism, Emotional Regulation did not statistically significantly relate to Study Engagement (*β*: 0.12, S.E.:0.06, *p* = 0.07), while optimism showed to have a direct positive relationship with Study Engagement (*β*: 0.49, S.E.:0.08, *p* < 0.05), and consisted of 29% of its total variance.

### 3.4. Assessing the Indirect Effects

This study’s final objective was to investigate the extent to which Emotional Regulation indirectly affected Stress, Anxiety, Depression, and Study Engagement through Optimism. It also aimed to determine whether optimism indirectly affected Depression through Study Engagement. As such, the BCB method of Preacher et al., 2011 with 10,000 iterations was employed to generate two-sided bias-corrected confidence intervals at the 95% marker (CI 95).

The results summarized in Table 5 show that a statistically significant (*p* < 0.05) indirect effect exists between Emotional regulation, Optimism and Study Engagement (*β*: 0.16, CI 95: lower 0.09, upper 0.27), Depression (*β*: −0.13, CI 95: lower −0.23, upper −0.27), and Anxiety (*β*: −0.08, CI 95: lower −0.17, upper −0.03). Furthermore, it was found that optimism indirectly affected Depression through Study Engagement (*β*: −0.12, CI 95: lower −0.20, upper −0.05). As the CIs range between for all these factors did not include zero, these indirect effects can be confirmed.

However, the results showed that Emotional Regulation did not indirectly affect Stress through Optimism (*p* > 0.05, CI 95: lower −0.14, upper 0.01). As both a non-significant indirect effect was found and the CI 95 range included zero, the indirect effect could not be established.

## 4. Discussion

The purpose of this study was (1) to document the current levels of mental health and study engagement of mental healthcare students in Tunisia during the COVID-19 pandemic spread, and (2) to investigate the relationships between emotional regulation, optimism, study engagement and common mental health problems (stress, anxiety and depression) among this population. A majority of the students reported mild (34.7%) or moderate (44.3%) levels of depression, moderate (44.7%) or severe (33.6%) levels of anxiety, and mild (33.8%) levels of stress, while they reported high or very high levels of study engagement (>85%). Furthermore, the results showed that high levels of emotional regulation and optimism negatively affected students’ experiences of stress, anxiety and depression. It further showed that higher levels of emotional regulation could lead to a more optimistic view of life, which in turn could increase study engagement. When students experienced higher levels of study engagement, the results showed that students also reported lower levels of depression. The study highlighted the importance of optimism as a mechanism that could translate higher levels of emotional regulation into lower levels of depression and anxiety. Finally, our results showed that when students had higher levels of optimism, they were more engaged in their studies, which negatively affected their levels of depression. In sum, these results highlighted the potential protective role of emotional regulation and optimism for students during the COVID-19 pandemic spread.

Previous research has found a high prevalence of psychological distress among university students, with a higher risk for healthcare students [16,17,18]. However, the current study results revealed that the levels of depression and anxiety symptoms in this sample were even higher than those reported in prior studies [19]. Our results show that stress, anxiety, and depression appear to be common within the current sample, which may negatively affect their academic performance. The high levels of stress in healthcare students may be due to academic stressors, including the nature of examination, long hours of study, lack of free time, and low faculty response to students needs without forgetting clinical sources of stress for healthcare students [20]. The higher levels of anxiety and depression symptoms in this study may be further fueled by the current COVID-19 pandemic. Healthcare students are particularly vulnerable to the onset of these common mental health problems during this time because of the fear of contracting the disease [68], an increased workload, and the greater pressure related to the challenges of making difficult moral decisions about healthcare priorities [69]. As the current pandemic appears to represent a specific risk factor that may not be directly controllable, it is all the more useful to identify potential protective factors during a situation of increased anxiety, such as the spread of COVID-19.

The current study results indicated that optimism and emotional regulation were found to independently and jointly negatively affect stress, anxiety and depression symptoms, and positively affect study engagement. These results are consistent with previous findings showing that optimism was positively related to mental health in university students [11,42,43]. These effects can also be explained by the fact that optimism and emotional regulation are related to higher perceived social support and to greater use of active coping strategies, including social support seeking (e.g., Brissette et al. [70]). Furthermore, the present study also indicated that optimism partially mediated the relationship between emotional regulation and anxiety and depression symptoms. Based on the broaden-and-build theory of positive emotions [71,72], this partial mediation can be explained by the fact that positive emotional experiences increase personal resources and vitality and creative problem-solving abilities, which help prevent mental health problems. Furthermore, research on positive emotions has shown that they broaden individuals’ thought and action repertoires and increase future time perspective [73]. This may explain how emotional regulation may lead to increased optimism—considered a positive future time perspective—which is related to reduced anxiety and depression symptoms, including in times of crisis such as a natural disaster [74].

Our study indicated that optimism fully mediated the relationship between emotional regulation and study engagement. This may be explained by the fact that emotional regulation competencies increase individual resources that can be engaged in actions directed towards long term goals (future diploma and profession) when positive expectancies regarding the future support this engagement. These positive emotional experiences lead to greater vitality and personal resources, increasing study engagement (e.g., Ouweneel et al. [75]).

Several studies have also shown how study engagement represents a significant predictor of academic achievement and student wellbeing [50]. Study engagement, which includes positive emotions such as inspiration, enthusiasm and pride, may have a similar effect on broadening thought and action repertoires, and increasing personal resources [50]. This may also explain our results, which showed that study engagement negatively affected depression symptoms. A further explanation can be found in the fact that study engagement is also related to meaningfulness [76], which is negatively related to depression symptoms [77]. This result is also in line with longitudinal data showing that work engagement is an antecedent of depression levels—measured three and four years later—rather than an outcome [78].

Although the results of the study highlight potential useful student mental health protection factors, some limitations need to be mentioned.First, the study sample was only recruited among healthcare students coming from one university in Tunisia. Hence, the results may not be generalized to other populations or countries. Second, the participants mainly consisted of women, and it has recently been shown that there are important gender differences in student mental health consequences of the COVID-19 pandemic [79,80,81,82]. This limits the generalizability of the results to the male population. Furthermore, other social and demographic factors such as religion and social support would need to be considered in future studies, as they may act as potential confounder factors among mental health protection factors.

While considering these limitations, based on the mental health protection factors identified through this study (i.e., emotional regulation and optimism), it would appear to be useful to develop and disseminate evidence-based interventions for college students in order to improve mental health through increased emotional regulation competencies and optimism, in particular for healthcare students.

One way of improving the accessibility of these interventions is through mobile phone apps or web-based programs, as they have been shown to be effective in reducing student stress, anxiety and depression [83,84,85,86,87,88], and improving wellbeing [89,90]. Indeed, as current students are considered digital natives, they are used to such highly accessible tools and show preferences toward self-help and using the Internet for many of their activities [91,92]. Among the advantages that have been underlined is the privacy of web-based interventions which may represent an interesting option compared to face-to-face or phone counselling. Indeed, past research has underlined difficulties for healthcare students to seek professional help, as it is not anonymous [86].

Among the effective interventions studied, positive psychology programs have been shown to be effective in improving mental health and academic engagement, notably through improved emotional regulation and optimism [93,94,95,96]. Mindfulness-based programs have also been shown to be effective on student stress [97] and psychological distress [98,99], which can notably be due to greater emotional regulation, as shown in school-based mindfulness programs [100]. Such programs could therefore also be useful for student populations, and are becoming popular, especially among medical schools [101]. Future studies should also assess the psychological processes and social and emotional competences which are developed through these interventions and how each of them relates to the specific characteristics of improved well-being. To this end, specific models of emotional intelligence have been developed to identify the various metacognitive and metaemotional skills that compose this dynamic set, and examples of strategies that can be used to develop these skills (e.g., Ref. [102]).

## 5. Conclusions

Students’ psychological distress is a global issue and has become even more important during the COVID-19 pandemic, in particular for healthcare students who were more exposed than other students to risks, disease and death. The findings of this study underlined that levels of anxiety and depression were more severe in this Tunisian healthcare student sample, which is in line with the COVID-19 pandemic situation. Additionally, this study identified two potential protection factors (optimism and emotional regulation), which may be targeted in prevention interventions, as they negatively affected depression and anxiety symptoms, and positively affected study engagement. As study engagement is widely recognized as leading to positive academic outcomes and wellbeing, and considering the high prevalence of anxiety and depression among Tunisian healthcare students, efforts that aim at improving their mental health should be highly encouraged, including through specific interventions aiming at enhancing emotional regulation competencies and optimism.

## Figures and Tables

**Table 1:** Demographic characteristics and descriptive statistics of depression, anxiety, stress and study engagement.

Participants’ Characteristics		Frequency	Percentage
Gender	Females	344	94
	Males	22	6
Accommodation	With Family	126	34.4
	Student Residence	161	44
	Rental with friends	65	17.8
	Rental alone	14	3.8
Marital status (%)	Single	360	98.6
	Married	5	1.4
Children	Yes	5	1.4
	No	361	98.6
Academic field	Podiatry	43	11.7
	Emergency Care	114	31.1
	Operating Instrumentation	77	21
	Paediatric care	96	26.2
	Research Masters	36	9.8
Year of enrolment	1st year	142	38.8
	2nd year	128	35
	3rd year	96	26.2
Repetition of academic year	No	363	99.2
	Yes	3	0.8
Depression	Normal (0–9)	*n* = 48	13.1
	Mild (10–13)	*n* = 127	34.7
	Moderate (14–20)	*n* = 162	44.3
	Severe (21–27)	*n* = 29	7.9
	Extremely severe (28+)	---	---
Anxiety	Normal (0–7)	*n* = 6	1.6
	Mild (8–9)	*n* = 38	10.4
	Moderate (10–14)	*n* = 160	43.7
	Severe (15–19)	*n* = 123	33.6
	Extremely severe (20+)	*n* = 39	10.7
Stress	Normal (0–14)	*n* = 186	50.8
	Mild (15–18)	*n* = 131	35.8
	Moderate (19–25)	*n* = 47	12.8
	Severe (26–33)	*n* = 2	0.5
	Extremely severe (34+)	---	---
Vigour	Very low ≤ 2	*n* = 8	2.2
	Low 2.01–3.25	*n* = 6	1.6
	Average 3.26–4.80	*n* = 20	5.5
	High 4.81–5.65	*n* = 25	6.8
	Very High ≥ 5.66	*n* = 307	83.9
Dedication	Very low ≤ 1.33	*n* = 13	3.6
	Low 1.34–2.90	*n* = 10	2.7
	Average 2.91–4.70	*n* = 25	6.8
	High 4.71–5.69	*n* = 9	2.5
	Very high ≥ 5.70	*n* = 309	84.4
Absorption	Very low ≤ 1.17	*n* = 8	2.2
	Low 1.18–2.33	*n* = 13	3.6
	Average 2.34–4.20	*n* = 23	6.3
	High 4.21–5.33	*n* = 18	4.9
	Very high ≥ 5.51	*n* = 304	83.1

**Table 2:** Model Fit Statistics and Criteria.

Fit Indices	Cut-Off Criterion	Sensitive to N	Penalty for Model Complexity
Absolute fit indices			
Chi-Square (χ^2^)	Lowest comparative value between measurement models	Yes	No
	Non-Significant Chi-Square (*p* > 0.01)		
Approximate Fit Indices			
Root-Means-Square Error of Approximation (RMSEA)	0.06 to 0.08 (Marginally Acceptable); 0.01 to 0.05 (Excellent)	No	Yes
	Non-Significant RMSEA (*p* > 0.01)		
	90% Confidence Interval Range should not include zero		
Standardized Root Mean Square Residual (SRMR)	0.06 to 0.08 (Marginally Acceptable); 0.01 to 0.05 (Excellent)	Yes	No
Incremental fit indices			
Comparative Fit Index (CFI)	0.90 to 0.95 (Marginally Acceptable Fit); 0.96 to 0.99 (Excellent)	No	No
Tucker-Lewis Index (TLI)	0.90 to 0.95 (Marginally Acceptable Fit); 0.96 to 0.99 (Excellent)	No	Yes
Akaike Information Criterion (AIC)	Lowest value in comparative measurement models	Yes	Yes
Bayes Information Criterion (BIC)	Lowest value in comparative measurement models	Yes	Yes

Adapted from Hu & Bentler [63].

**Table 3:** Descriptive Statistics, Composite Reliability and Pearson’s Correlations.

No	Factor	Mean	SD	Skewness	Kurtosis	ρ	1	2	3	4	5	6	7	8
1	Stress	2.11	0.46	0.38	0.29	0.77	-							
2	Depression	1.99	0.56	0.58	−0.06	0.88	0.71	-	-	-	-	-	-	-
3	Anxiety	2.03	0.56	0.41	−0.16	0.85	0.87	0.76	-	-	-	-	-	-
4	Vigour	3.84	1.50	0.06	−0.77	0.71	−0.21	−0.49	−0.27	-	-	-	-	-
5	Dedication	4.62	1.56	−0.50	−0.54	0.79	−0.21	−0.50	−0.27	0.89	-	-	-	-
6	Absorption	4.29	1.41	−0.19	−0.54	0.65	−0.21	−0.50	−0.27	0.89	0.91	-	-	-
7	Overall Study Engagement	4.25	1.30	−0.18	−0.60	0.96	−0.22	−0.52	−0.29	0.93	0.95	0.95	-	-
8	Optimism	3.44	0.70	−0.29	0.02	0.61	−0.33	−0.61	−0.39	0.49	0.50	0.50	0.53	-
9	Emotion Regulation	3.44	0.92	−0.36	−0.32	0.74	−0.53	−0.46	−0.41	0.26	0.27	0.27	0.28	0.33

All factors were statistically significantly related at *p* < 0.05.

**Table 4:** Measurement Model Fit Statistics.

Model	χ^2^	*p*-Value	df	χ^2^/df	CFI	TLI	RMSEA	SRMR	OFV	90% C.I RMSEA		Meets Criteria
										LL	UL	
Model 1	968.72	0.00	649	1.49	0.93	0.93	0.04	0.05	0.11	0.032	0.041	Yes
Model 2	1169.58	0.00	688	1.70	0.90	0.89	0.04	0.06	0.15	0.039	0.048	No
Model 3	1338.01	0.00	694	1.93	0.87	0.86	0.05	0.07	0.18	0.046	0.054	No
Model 4	1153.74	0.00	703	1.64	0.90	0.89	0.05	0.06	0.15	0.041	0.050	No
Model 5	1122.60	0.00	667	1.68	0.90	0.89	0.04	0.06	0.15	0.039	0.048	No

**Table 5:** Indirect Effects Estimation.

Variable	Estimate	S.E.	*p*-Value	95% BC CI	Indirect Effect Present
Emotional regulation indirectly affects engagement through Optimism	0.16	0.05	0.00	[0.09; 0.27]	Yes
Emotional regulation indirectly affects stress through Optimism	−0.06	0.04	0.09	[−0.14; 0.01]	No
Emotional regulation indirectly affects depression through Optimism	−0.13	0.04	0.00	[−0.23; −0.06]	Yes
Emotional regulation indirectly affects anxiety through Optimism	−0.08	0.04	0.02	[−0.17; −0.03]	Yes
Optimism indirectly affects depression through Engagement	−0.12	0.04	0.00	[−0.20; −0.05]	Yes

## Data Availability

Data is available upon request to the corresponding author.

## Abbreviations

PEC	Profile of Emotional Competence
LOT-R	Life Orientation Test-Revised
UWES	Utrecht Work Engagement Scale
DAS-21	Depression, Anxiety and Stress Scale-21

## References

[1] Chen R.-N., Liang S.-W., Peng Y., Li X.-G., Chen J.-B., Tang S.-Y., Zhao J.-B. (2020). Mental health status and change in living rhythms among college students in China during the COVID-19 pandemic: A large-scale survey. J. Psychosom. Res..

[2] Khan A.H., Sultana M.S., Hossain S., Hasan M.T., Ahmed H.U., Sikder T. (2020). The impact of COVID-19 pandemic on mental health & wellbeing among home-quarantined Bangladeshi students: A cross-sectional pilot study. J. Affect. Disord..

[3] Salman M., Asif N., Mustafa Z.U., Khan T.M., Shehzadi N., Hussain K., Tahir H., Raza M.H., Khan M.T. (2020). Psychological Impact of COVID-19 on Pakistani University Students and How They Are Coping. Medrxiv.

[4] Van Zyl L.E. (2021). Social Study Resources and Social Wellbeing Before and During the Intelligent COVID-19 Lockdown in The Netherlands. Soc. Indic. Res..

[5] Van Zyl L.E., Rothmann S., Zondervan-Zwijnenburg M.A.J. (2021). Longitudinal Trajectories of Study Characteristics and Mental Health Before and During the COVID-19 Lockdown. Front. Psychol..

[6] Sun S., Goldberg S.B., Lin D., Qiao S., Operario D. (2021). Psychiatric symptoms, risk, and protective factors among university students in quarantine during the COVID-19 pandemic in China. Glob. Health.

[7] Auerbach R.P., Alonso J., Axinn W.G., Cuijpers P., Ebert D.D., Green J.G., Hwang I., Kessler R.C., Liu H., Mortier P. (2016). Mental disorders among college students in the World Health Organization World Mental Health Surveys. Psychol. Med..

[8] Tang F., Byrne M., Qin P. (2018). Psychological distress and risk for suicidal behavior among university students in contemporary China. J. Affect. Disord..

[9] Larcombe W., Finch S., Sore R., Murray C.M., Kentish S., Mulder R.A., Lee-Stecum P., Baik C., Tokatlidis O., Williams D.A. (2014). Prevalence and socio-demographic correlates of psychological distress among students at an Australian university. Stud. High. Educ..

[10] Shankland R., Genolini C., Franca L.R., Guelfi J.-D., Ionescu S. (2009). Student adjustment to higher education: The role of alternative educational pathways in coping with the demands of student life. High. Educ..

[11] Saleh D., Camart N., Romo L. (2017). Predictors of Stress in College Students. Front. Psychol..

[12] Baghurst T., Kelley B.C. (2014). An Examination of Stress in College Students Over the Course of a Semester. Health Promot. Pract..

[13] Conley C.S., Ma L.V.T., Bryant F.B. (2013). Promoting Psychosocial Adjustment and Stress Management in First-Year College Students: The Benefits of Engagement in a Psychosocial Wellness Seminar. J. Am. Coll. Health.

[14] Shankland R., Kotsou I., Vallet F., Bouteyre E., Dantzer C., Leys C. (2019). Burnout in university students: The mediating role of sense of coherence on the relationship between daily hassles and burnout. High. Educ..

[15] Houghton J.D., Wu J., Godwin J.L., Neck C.P., Manz C.C. (2012). Self-Leadership, and Student Stress Coping. J. Manag. Educ..

[16] Dyrbye L.N., Thomas M.R., Shanafelt T.D. (2006). Systematic Review of Depression, Anxiety, and Other Indicators of Psychological Distress Among, U.S. and Canadian Medical Students. Acad. Med..

[17] Dyrbye L.N., Thomas M.R., Massie F.S., Power D.V., Eacker A., Harper W., Durning S., Moutier C., Szydlo D.W., Novotny P.J. (2008). Burnout and Suicidal Ideation among U.S. Medical Students. Ann. Intern. Med..

[18] Kumar H. (2016). Psychological Distress and Life Satisfaction among University Students. J. Psychol. Clin. Psychiatry.

[19] Nacht J. (2016). Prevalence of Depression and Depressive Symptoms Among Resident Physicians: A Systematic Review and Meta-analysis. J. Emerg. Med..

[20] Papazisis G., Papanikolaou N., Vlasiadis I., Sapountzi-Krepia D. (2008). Psychological Distress, Anxiety and Depression among Nursing Students in Greece. Int. J. Caring Sci..

[21] Pulido-Martos M., Augusto-Landa J., Lopez-Zafra E. (2011). Sources of stress in nursing students: A systematic review of quantitative studies. Int. Nurs. Rev..

[22] Chan C.K., So W.K., Fong D.Y. (2009). Hong Kong Baccalaureate Nursing Students’ Stress and Their Coping Strategies in Clinical Practice. J. Prof. Nurs..

[23] Lo R. (2002). A longitudinal study of perceived level of stress, coping and self-esteem of undergraduate nursing students: An Australian case study. J. Adv. Nurs..

[24] Galbraith N.D., Brown K.E. (2011). Assessing intervention effectiveness for reducing stress in student nurses: Quantitative systematic review. J. Adv. Nurs..

[25] Marzouk M., Ouanes-Besbes L., Ouanes I., Hammouda Z., Dachraoui F., Abroug F. (2018). Prevalence of anxiety and depressive symptoms among medical residents in Tunisia: A cross-sectional survey. BMJ Open.

[26] Kwok S.Y.C.L., Gu M. (2017). The Role of Emotional Competence in the Association Between Optimism and Depression Among Chinese Adolescents. Child Indic. Res..

[27] Shaheen H., Jahan M. (2014). The Role of Optimism in experience of Student Stress and Suicidal Ideation. IOSR J. Humanit. Soc. Sci..

[28] Gross J.J. (2002). Emotion regulation: Affective, cognitive, and social consequences. Psychophysiology.

[29] Lopes P.N., Brackett M.A., Nezlek J., Schütz A., Sellin I., Salovey P. (2004). Emotional Intelligence and Social Interaction. Pers. Soc. Psychol. Bull..

[30] Lopes P.N., Salovey P., Côté S., Beers M. (2005). Emotion Regulation Abilities and the Quality of Social Interaction. Emotion.

[31] Lopes P.N., Salovey P., Straus R. (2003). Emotional intelligence, personality, and the perceived quality of social relationships. Pers. Individ. Differ..

[32] Capone V., Caso D., Donizzetti A., Procentese F. (2020). University Student Mental Well-Being during COVID-19 Outbreak: What Are the Relationships between Information Seeking, Perceived Risk and Personal Resources Related to the Academic Context?. Sustainability.

[33] Cilliers J., Mostert K., Nel J. (2017). Study demands, study resources and the role of personality characteristics in predicting the engagement of fist-year university students. S. Afr. J. High. Educ..

[34] Lea R., Davis S., Mahoney B., Qualter P. (2019). Does Emotional Intelligence Buffer the Effects of Acute Stress? A Systematic Review. Front. Psychol..

[35] Drigas A., Papoutsi C. (2020). The Need for Emotional Intelligence Training Education in Critical and Stressful Situations: The Case of COVID-19. Int. J. Recent Contrib. Eng. Sci. IT.

[36] Gross J.J. (1998). The Emerging Field of Emotion Regulation: An Integrative Review. Rev. Gen. Psychol..

[37] Gross J.J. (2015). Emotion Regulation: Current Status and Future Prospects. Psychol. Inq..

[38] Tugade M.M., Fredrickson B.L. (2007). Regulation of Positive Emotions: Emotion Regulation Strategies that Promote Resilience. J. Happiness Stud..

[39] Ashby F.G., Isen A.M., Turken A.U. (1999). A neuropsychological theory of positive affect and its influence on cognition. Psychol. Rev..

[40] Tugade M.M., Fredrickson B.L. (2004). Resilient Individuals Use Positive Emotions to Bounce Back from Negative Emotional Experiences. J. Pers. Soc. Psychol..

[41] Vizoso C., Arias-Gundín O., Rodríguez C. (2019). Exploring coping and optimism as predictors of academic burnout and performance among university students. Educ. Psychol..

[42] Burris J.L., Brechting E.H., Salsman J., Carlson C.R. (2009). Factors Associated with the Psychological Well-Being and Distress of University Students. J. Am. Coll. Health.

[43] Scheier M.F., Carver C. (1992). Effects of optimism on psychological and physical well-being: Theoretical overview and empirical update. Cogn. Ther. Res..

[44] Gómez-Molinero R., Zayas A., Ruíz-González P., Guil R. (2018). Optimism and resilience among university students. Int. J. Dev. Ment. Educ. Psychol. Rev. INFAD Psicol..

[45] Peterson C., Steen T.A., Snyder C.R., Lopez S.J. (2002). Optimistic Explanatory Style. Handbook of Positive Psychology.

[46] Lesener T., Pleiss L.S., Gusy B., Wolter C. (2020). The Study Demands-Resources Framework: An Empirical Introduction. Int. J. Environ. Res. Public Health.

[47] Schaufeli W.B., Salanova M., González-Romá V., Bakker A.B. (2002). The Measurement of Engagement and Burnout: A Two Sample Confirmatory Factor Analytic Approach. J. Happiness Stud..

[48] Schaufeli W.B., Martinez I.M.M., Pinto A.M., Salanova M., Bakker A.B. (2002). Burnout and Engagement in University Students. J. Cross-Cult. Psychol..

[49] Schaufeli W.B. (2017). General Engagement: Conceptualization and Measurement with the Utrecht General Engagement Scale (UGES). J. Well-Being Assess..

[50] Salanova M., Schaufeli W., Martinez I.M.M., Breso E. (2010). How obstacles and facilitators predict academic performance: The mediating role of study burnout and engagement. AnxietyStress Coping.

[51] Tayama J., Schaufeli W., Shimazu A., Tanaka M., Takahama A. (2019). Validation of a Japanese Version of the Work Engagement Scale for Students. Jpn. Psychol. Res..

[52] Brasseur S., Grégoire J., Bourdu R., Mikolajczak M. (2013). The Profile of Emotional Competence (PEC): Development and Validation of a Self-Reported Measure that Fits Dimensions of Emotional Competence Theory. PLoS ONE.

[53] Segerstrom S.C., Evans D.R., Eisenlohr-Moul T.A. (2011). Optimism and pessimism dimensions in the Life Orientation Test-Revised: Method and meaning. J. Res. Pers..

[54] Schaufeli W.B., Bakker A.B., Salanova M. (2006). The Measurement of Work Engagement with a Short Questionnaire: A Cross-National Study. Educ. Psychol. Meas..

[55] Schaufeli W.B., Bakker A.B. (2003). UWES-Utrecht Work Engagement Scale: Test Manual.

[56] Lovibond P.F., Lovibond S.H. (1995). The structure of negative emotional states: Comparison of the Depression Anxiety Stress Scales (DASS) with the Beck Depression and Anxiety Inventories. Behav. Res. Ther..

[57] Coker A., Coker O., Sanni D. (2018). Psychometric properties of the 21-item Depression Anxiety Stress Scale (DASS-21). Afr. Res. Rev..

[58] IBM (2019). SPSS Statistics for Windows.

[59] Muthén L.K., Muthén B.O. (2020). Mplus User’s Guide.

[60] Kim H.-Y. (2013). Statistical notes for clinical researchers: Assessing normal distribution (2) using skewness and kurtosis. Restor. Dent. Endod..

[61] Raykov T. (2009). Evaluation of Scale Reliability for Unidimensional Measures Using Latent Variable Modeling. Meas. Evaluation Couns. Dev..

[62] Brown T.A. (2006). Confirmatory Factor Analysis for Applied Research. Methodology in the Social Sciences.

[63] Hu L.-T., Bentler P.M. (1999). Cutoff criteria for fit indexes in covariance structure analysis: Conventional criteria versus new alternatives. Struct. Equ. Model. A Multidiscip. J..

[64] McNeish D., Hancock G.R. (2018). The effect of measurement quality on targeted structural model fit indices: A comment on Lance, Beck, Fan, and Carter (2016). Psychol. Methods.

[65] Preacher K.J., Zhang Z., Zyphur M. (2011). Alternative Methods for Assessing Mediation in Multilevel Data: The Advantages of Multilevel SEM. Struct. Equ. Model. A Multidiscip. J..

[66] Asparouhov T., Muthén B. (2009). Exploratory Structural Equation Modeling. Struct. Equ. Model. A Multidiscip. J..

[67] Kline B. (2010). Principles and Practice of Structural Equation Modeling.

[68] Alnazly E., Khraisat O.M., Al-Bashaireh A.M., Bryant C.L. (2021). Anxiety, depression, stress, fear and social support during COVID-19 pandemic among Jordanian healthcare workers. PLoS ONE.

[69] Sahebi A., Nejati-Zarnaqi B., Moayedi S., Yousefi K., Torres M., Golitaleb M. (2021). The prevalence of anxiety and depression among healthcare workers during the COVID-19 pandemic: An umbrella review of meta-analyses. Prog. Neuro-Psychopharmacol. Biol. Psychiatry.

[70] Brissette I., Scheier M.F., Carver C.S. (2002). The role of optimism in social network development, coping, and psychological adjustment during a life transition. J. Pers. Soc. Psychol..

[71] Fredrickson B.L. (1998). What Good Are Positive Emotions?. Rev. Gen. Psychol..

[72] Fredrickson B.L. (2001). The Role of Positive Emotions in Positive Psychology. Am. Psychol..

[73] Denovan A., Dagnall N., Macaskill A., Papageorgiou K. (2019). Future time perspective, positive emotions and student engagement: A longitudinal study. Stud. High. Educ..

[74] Van der Velden P.G., Kleber R.J., Fournier M., Grievink L., Drogendijk A., Gersons B.P. (2007). The association between dispositional optimism and mental health problems among disaster victims and a comparison group: A prospective study. J. Affect. Disord..

[75] Ouweneel E., Le Blanc P.M., Schaufeli W.B. (2011). Flourishing students: A longitudinal study on positive emotions, personal resources, and study engagement. J. Posit. Psychol..

[76] Van Zyl L.E., Deacon E., Rothmann S. (2010). Towards happiness: Experiences of work-role fit, meaningfulness and work engagement of industrial/organisational psychologists in South Africa. SA J. Ind. Psychol..

[77] Mascaro N., Rosen D.H. (2005). Existential Meaning’s Role in the Enhancement of Hope and Prevention of Depressive Symptoms. J. Pers..

[78] Hakanen J.J., Schaufeli W.B. (2012). Do burnout and work engagement predict depressive symptoms and life satisfaction? A three-wave seven-year prospective study. J. Affect. Disord..

[79] Ausín B., González-Sanguino C., Castellanos M.Á., Muñoz M. (2020). Gender-related differences in the psychological impact of confinement as a consequence of COVID-19 in Spain. J. Gend. Stud..

[80] Barros C., Sacau-Fontenla A. (2021). New Insights on the Mediating Role of Emotional Intelligence and Social Support on University Students’ Mental Health during COVID-19 Pandemic: Gender Matters. Int. J. Environ. Res. Public Health.

[81] Batra K., Sharma M., Batra R., Singh T., Schvaneveldt N. (2021). Assessing the Psychological Impact of COVID-19 among College Students: An Evidence of 15 Countries. Healthcare.

[82] Prowse R., Sherratt F., Abizaid A., Gabrys R.L., Hellemans K.G., Patterson Z.R., McQuaid R.J. (2021). Coping with the COVID-19 Pandemic: Examining Gender Differences in Stress and Mental Health Among University Students. Front. Psychiatry.

[83] Harrer M., Apolinário-Hagen J., Fritsche L., Drüge M., Krings L., Beck K., Salewski C., Zarski A.-C., Lehr D., Baumeister H. (2019). Internet- and App-Based Stress Intervention for Distance-Learning Students with Depressive Symptoms: Protocol of a Randomized Controlled Trial. Front. Psychiatry.

[84] Hintz S., Frazier P.A., Meredith L. (2015). Evaluating an online stress management intervention for college students. J. Couns. Psychol..

[85] Lintvedt O.K., Griffiths K.M., Sørensen K., Østvik A.R., Wang C.E.A., Eisemann M., Waterloo K. (2011). Evaluating the effectiveness and efficacy of unguided internet-based self-help intervention for the prevention of depression: A randomized controlled trial. Clin. Psychol. Psychother..

[86] Nguyen-Feng V.N., Frazier P.A., Greer C.S., Howard K.G., Paulsen J.A., Meredith L., Kim S. (2015). A randomized controlled trial of a web-based intervention to reduce distress among students with a history of interpersonal violence. Psychol. Violence.

[87] Orbach G., Lindsay S., Grey S. (2007). A randomised placebo-controlled trial of a self-help Internet-based intervention for test anxiety. Behav. Res. Ther..

[88] Saleh D., Camart N., Sbeira F., Romo L. (2018). Can we learn to manage stress? A randomized controlled trial carried out on university students. PLoS ONE.

[89] George D.R., Dellasega C., Whitehead M.M., Bordon A. (2013). Facebook-based stress management resources for first-year medical students: A multi-method evaluation. Comput. Hum. Behav..

[90] Harrer M., Adam S.H., Fleischmann R.J., Baumeister H., Auerbach R., Bruffaerts R., Cuijpers P., Kessler R.C., Berking M., Lehr D. (2018). Effectiveness of an Internet- and App-Based Intervention for College Students with Elevated Stress: Randomized Controlled Trial. J. Med. Internet Res..

[91] Davies E.B., Morriss R., Glazebrook C., Gulliver A., Spates C. (2014). Computer-Delivered and Web-Based Interventions to Improve Depression, Anxiety, and Psychological Well-Being of University Students: A Systematic Review and Meta-Analysis. J. Med. Internet Res..

[92] Day V., McGrath P., Wojtowicz M. (2013). Internet-based guided self-help for university students with anxiety, depression and stress: A randomized controlled clinical trial. Behav. Res. Ther..

[93] Baños R.M., Etchemendy E., Farfallini L., García-Palacios A., Quero S., Botella C. (2014). EARTH of Well-Being System: A pilot study of an Information and Communication Technology-based positive psychology intervention. J. Posit. Psychol..

[94] Hammill J., Nguyen T., Henderson F. (2020). Student engagement: The impact of positive psychology interventions on students. Act. Learn. High. Educ..

[95] Machado L., De Oliveira I.R., Peregrino A., Cantilino A. (2019). Common mental disorders and subjective well-being: Emotional training among medical students based on positive psychology. PLoS ONE.

[96] Sitbon A., Shankland R., Krumm C.-M. (2019). Interventions efficaces en psychologie positive: Une revue systématique. Can. Psychol. Can..

[97] Regehr C., Glancy D., Pitts A. (2013). Interventions to reduce stress in university students: A review and meta-analysis. J. Affect. Disord..

[98] De Vibe M., Solhaug I., Tyssen R., Friborg O., Rosenvinge J.H., Sørlie T., Bjørndal A. (2013). Mindfulness training for stress management: A randomised controlled study of medical and psychology students. BMC Med. Educ..

[99] Galante J., Dufour G., Benton A., Howarth E., Vainre M., Croudace T., Wagner A.P., Stochl J., Jones P.B. (2016). Protocol for the Mindful Student Study: A randomised controlled trial of the provision of a mindfulness intervention to support university students’ well-being and resilience to stress. BMJ Open.

[100] Theurel A., Gimbert F., Gentaz E. (2018). Quels sont les bénéfices académiques, cognitifs, socio-émotionnels et psychologiques des interventions basées sur la pleine conscience en milieu scolaire?. Une Synthèse De.

[101] Barnes N., Hattan P., Black D.S., Schuman-Olivier Z. (2017). An examination of mindfulness-based programs in U.S. medical schools. Mindfulness.

[102] Drigas A., Papoutsi C., Skianis C. (2021). Metacognitive and Metaemotional Training Strategies through the Nine-layer Pyramid Model of Emotional Intelligence. Int. J. Recent Contrib. Eng. Sci. IT.

